# Lactate clearance is associated with mortality in septic patients with acute kidney injury requiring continuous renal replacement therapy: A cohort study

**DOI:** 10.1097/MD.0000000000005112

**Published:** 2016-10-07

**Authors:** Rogério da Hora Passos, Joao Gabriel Rosa Ramos, André Gobatto, Evandro José Bulhões Mendonça, Eva Alves Miranda, Fábio Ricardo Dantas Dutra, Maria Fernanda R Coelho, Andrea C Pedroza, Paulo Benigno Pena Batista, Margarida Maria Dantas Dutra

**Affiliations:** a Critical Care Unit Hospital Sao Rafael, Salvador, Brazil /Nephrology Division Hospital Portugues, Salvador; b Critical Care Unit Hospital São Rafael, Salvador, Brazil/UNIME Medical School, Lauro de Freitas; c Critical Care Unit Hospital São Rafael; d Nephrology Division, Hospital Português, Salvador; e Hospital São Rafael Critical Care Unit Hospital São Rafael, Salvador, Brazil/UNIME Medical School, Lauro de Freitas, Brazil.

**Keywords:** acidosis, acute kidney injury, CVVDF, lactate, lactate clearance, outcome, sepsis

## Abstract

The aim of the study was to assess the clinical utility of lactate measured at different time points to predict mortality at 48 hours and 28 days in septic patients with acute kidney injury (AKI) requiring continuous renal replacement therapy (CRRT).

Consecutive critically ill patients with septic AKI requiring CRRT were prospectively studied. Variables were collected at initiation of CRRT and 24 hours later.

In total, 186 patients were analyzed. Overall mortality at 48 hours was 28% and at 28 days was 69%. Initial lactate, lactate at 24 hours and the proportion of patients with a lactate clearance superior to 10% were different between survivors at 28 days [2.0 mmol/L, 1.95 mmol/L and 18/45 (40%)] and nonsurvivors [3.46 mmol, 4.66 mmol, and 18/94 (19%)]. Multivariate analysis demonstrated that lactate at 24 hours and lactate clearance, but not initial lactate, were independently associated to mortality. Area under the ROC curves for 28-day mortality was 0.635 for initial lactate; 0.828 for lactate at 24 hours and 0.701 for lactate clearance.

Lactate clearance and lactate after 24 hours of CRRT, but not initial lactate, were independently associated with mortality in septic AKI patients undergoing CRRT. Serial lactate measurements may be useful prognostic markers than initial lactate in these patients.

## 1 Introduction

Septic acute kidney injury is the dominant cause of acute kidney injury (AKI) and it confers an independent increase in risk for intensive care unit (ICU) morbidity and mortality.^[1]^ Despite substantial progress in the understanding of mechanisms involved, there is still a pool of questions preclusive of the development of effective therapeutic strategies.^[2,1,3]^ Septic AKI is characterized by greater severity of illness as demonstrated by higher severity scores, greater aberrancy in vital signs and need for organ support therapy.^[1,4]^ Patients with AKI and septic shock are particularly suited to continuous renal replacement therapy (CRRT), mostly continuous venous–venous hemodiafiltration (CVVHDF), because it is hemodynamically better tolerated and allows continuous fluid balance control.^[5]^

It has been shown that persistent acidosis during this procedure is a strong predictor of poor outcome.^[6]^ CRRT corrects metabolic acidosis through its effect on unmeasured anions, phosphate, and chloride.^[7]^ In patients with septic AKI the lack of improvement of acidosis on CRRT is associated with increased lactate levels, circulatory failure, and high mortality.^[8]^ Lactate clearance early in the hospital course may indicate a resolution of global tissue hypoxia and is associated with decreased mortality rate in patients with severe sepsis and septic shock,^[9]^ so lactate clearance has been used as one of the goals of early sepsis resuscitation;^[10]^ however, it is not clear if these data apply to septic patients in need of CRRT.

Accordingly, in view of the limited data on septic AKI requiring CRRT, we sought to assess the clinical utility of lactate level measured at initiation of CRRT, lactate measured at 24 hours after start of CRRT and lactate clearance as predictive factors of early (48 hours) and late (28 days) mortality in critically ill patients with septic AKI on CRRT.

## 2 Material and methods

### 2.1 Consent and ethics

The study was approved by the ethics committee of Hospital Portugues (Salvador, Brazil). Because routine collection of data entered into the database did not modify patients’ management in any way, and statistical analyses were processed anonymously, informed consent for participation in the study was waived.

### 2.2 Study design

All consecutive patients with septic AKI requiring CRRT admitted in 3 different intensive care units (cardiac, medical, and surgical units) at Hospital Portugues (a Brazilian tertiary hospital) between October 2005 and December of 2010 were prospectively studied. In the event of multiple ICU admissions, only the initial ICU admission was considered.

AKI was classified according to the Acute Kidney Injury Network (AKIN) criteria. Baseline serum creatinine values were estimated using the Modification of Diet in Renal Disease (MDRD) equation, as recommended by the Acute Dialysis Quality Initiative (ADQI) Working Group (assuming a lower limit of normal baseline glomerular filtration rate of 75 mL/minute) and similar to previous studies.^[11]^ Sepsis was defined with the presence of a suspected infection combined with 2 of the following conditions, temperature >38 ^o^C or <36 ^o^C, heart rate >90 beats/min, respiratory rate >20 breaths/min or mechanical ventilation and with blood cell count >12 × 10 9/L or <4 × 10 9/L.^[12]^ Septic AKI was defined as simultaneous presence of both syndromes sepsis and AKI in the absence of other clear and established, nonsepsis-related precipitants of AKI (i.e., urinary tract obstruction, radio contrast media other nephrotoxins).^[2]^ Patients were followed through discharge or death. Primary outcome was defined as mortality at 28 days (late mortality). Early mortality (i.e., mortality at 48 hours) was also evaluated.

### 2.3 Data collection

Standard demographic, clinical, and laboratorial data were collected at the initiation of CRRT and 24 hours later. Arterial lactate was collected before CRRT initiation and 24 hours after CRRT initiation. Lactate clearance was defined using the following formula: lactate at CRRT initiation minus lactate at 24 hours after CRRT initiation, divided by lactate at CRRT initiation, then multiplied by 100. A positive value denotes a decrease or clearance of lactate, whereas a negative value denotes an increase in lactate after 24 hours of CVVHDF.^[9]^ Lactate clearance was further dichotomized in lactate clearance superior or inferior to 10%, because it is the value most often cited in the literature as associated with outcomes.^[9,10]^

Severity of illness was assessed using the Acute Physiology and Chronic Health Evaluation (APACHE) II^[13]^ and Sequential Organ Failure Assessment (SOFA) score.^[14]^ Organ failure (e.g., hepatic failure) was defined as a SOFA specific organ system score ≥2.

### 2.4 CRRT procedure

CVVHDF was the CRRT modality of choice and was performed by consulting nephrologists on the basis of standard clinical guidelines, including AKI with hemodynamic instability, ongoing hypercatabolism, hyperkalemia, severe acidosis, volume overload, respiratory distress, multiorgan failure, or some combination of these factors. Saline flushes were used to warrant filter patency, instead of heparin, as per institutional protocol. Patients were routinely treated by CVVHDF with bicarbonate buffered solution. The procedure was performed using the Gambro PRISMA CRRT machine. In all patients a M100 hemofilter was used and were routinely changed after 72 hours. The ultrafiltrate flow rate was set accordingly daily fluid balance gains. The prescribed dialysis dose was around 20 to 30 mL/kg/h.

### 2.5 Statistical analysis

Analysis was performed using IBM SPSS Statistics 18.0 Version (SPSS Inc.). Sample size was calculated as 168 patients, considering a power of 80%, an alpha of 0.05, a ratio of 4:1 of patients with a lactate clearance superior to 10%, a 28-day mortality rate of 50%, and an odds ratio of 0.3 for patients with lactate clearance superior to 10%. These prevalences and effects sizes were estimated from previous studies.^[15,9]^ Continuous variables were reported as means and standard deviations and compared using analysis of variance. Categorical data were reported as number (percentage) and compared using chi-square or Fisher's exact test, as appropriate.

Multivariable logistic regression analysis was used to control for confounders. The variables selected were those found to be significantly correlated to mortality in the univariate analysis and with clinical plausibility. Logistic regression was chosen as the multivariate method because, despite initial lactate and lactate after 24 hours being repeated measures of the same variable, the model was thought to be a prognostic model, and not a causal one. Furthermore, the value of the lactate at 24 hours did not show significant collinearity (as evaluated by tolerance test or variation inflation factor) with initial lactate and is probably influenced by other variables, besides initial lactate. Lactate clearance was analyzed as a *dummy* dichotomous variable (lactate clearance superior or inferior to 10%). Because lactate clearance was thought to be representative of the same similar biological processes of lactate at 24 hours and demonstrated correlation with lactate at 24 hours and initial lactate (as measured by the Pearson correlation matrix), different models were developed to evaluate the association of lactate clearance with mortality. Different models were developed for early (48 hours) and late (28 days) mortality. The final covariate models were developed by a stepwise procedure with backward elimination using Wald statistic. Probability for stepwise entry was 0.05 and removal was 0.10. Goodness-of-fit was tested by the Hosmer and Lemeshow statistic.

The prognostic value of initial lactate, lactate after 24 hours and lactate clearance was also evaluated by the analysis of the area under the receiver operator characteristic (ROC) curve. Data are presented with 95% confidence intervals (CIs) and a bicaudal *P* < 0.05 was considered statistically significant for all comparisons.

## 3 Results

### 3.1 Characteristics of the cohort

A total of 186 septic acute kidney injury patients undergoing CVVHDF were enrolled. Baseline characteristics of these patients are included in Table 1. For the overall sample the mean lactate before initiation of CRRT was 3.01 ± 2.93 and at 24 hours was 3.78 ± 3.1. Thirty-six (19.4%) of patients had a lactate clearance superior to 10%. Fifty-two (28%) and 129 (69%) of patients were dead at 48 hours and 28 days, respectively (Table 1).

**Table 1:**
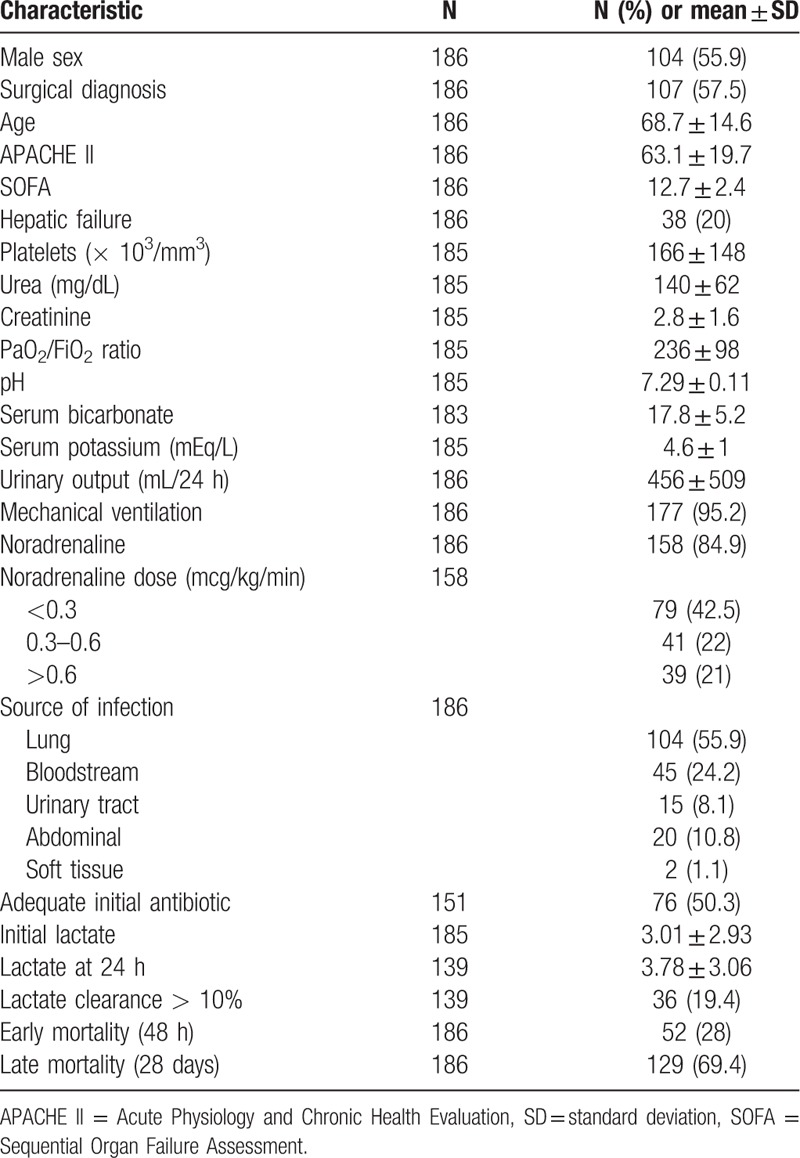
Baseline characteristics of the cohort.

### 3.2 Mortality analysis and association to lactate levels

Variables associated to early and late mortality are shown in Table 2. Mean initial lactate, lactate at 24 hours and lactate clearance were significantly different between survivors and nonsurvivors. A lactate clearance superior to 10% was associated to a reduced mortality [OR (95%CI) = 0.143 (0.032–0.634) for early mortality and OR (95%CI) = 0.355 (0.162–0.781) for late mortality].

**Table 2:**
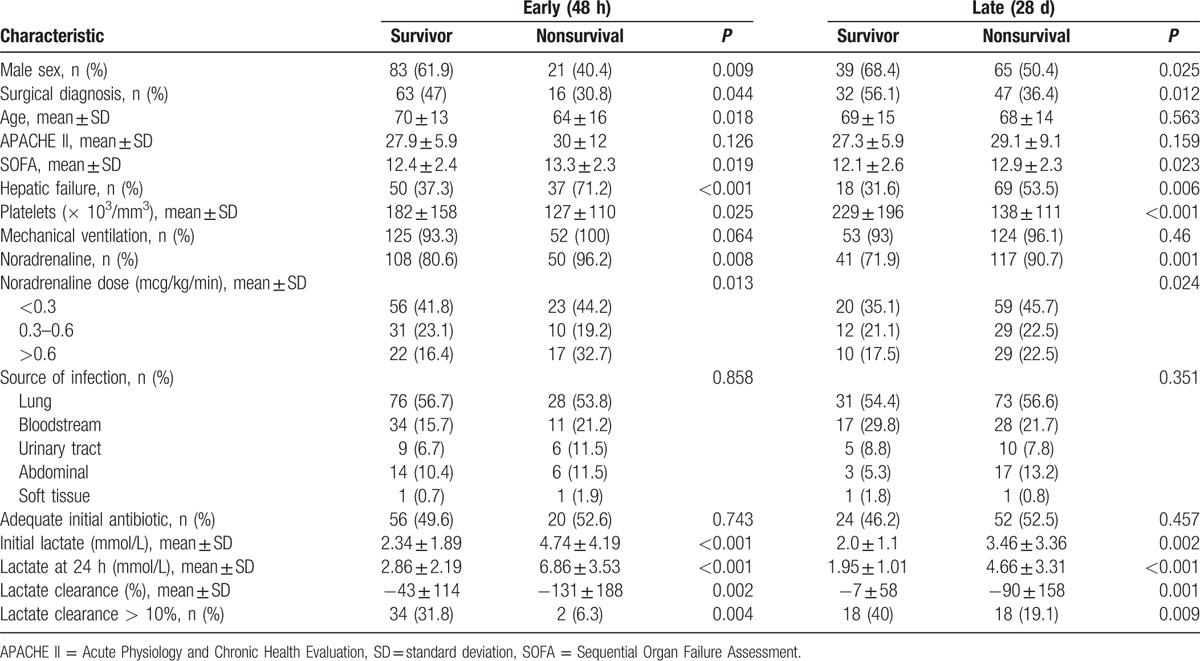
Univariate analysis for early (48 h) and late (28 d) mortality.

After adjusting for confounders, lactate at 24 hours after initiation of CRRT was significantly associated to early [OR (95%CI) = 1.72 (1.39–2.12)] and late [OR (95%CI) = 2.35 (1.57–3.51)] mortality (Table 3Tables 3A and B). Initial lactate, however, was not independently associated to mortality after multivariate analysis.

**Table 3:**
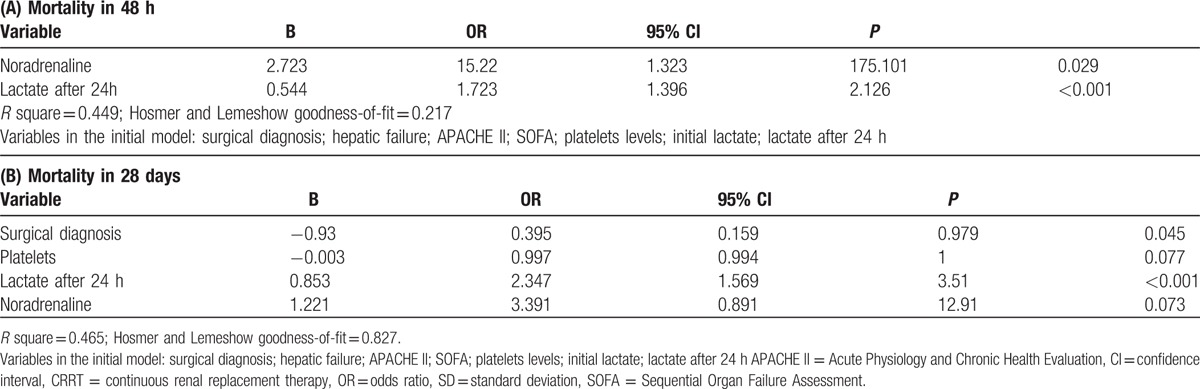
Multivariate analysis for early (A) and late (B) mortality. In these models, lactate was evaluated as initial lactate and lactate after 24 hours of initiation of CRRT.

Lactate clearance was evaluated in separate models (Table 4A and B), because it was highly correlated to both initial lactate and lactate after 24 hours. After adjusting for confounders, a lactate clearance superior to 10% was independently associated to a lower early [OR (95%CI) = 0.114 (0.025–0.527)] and late [OR (95%CI) = 0.235 (0.089–0.615)] mortality.

**Table 4:**
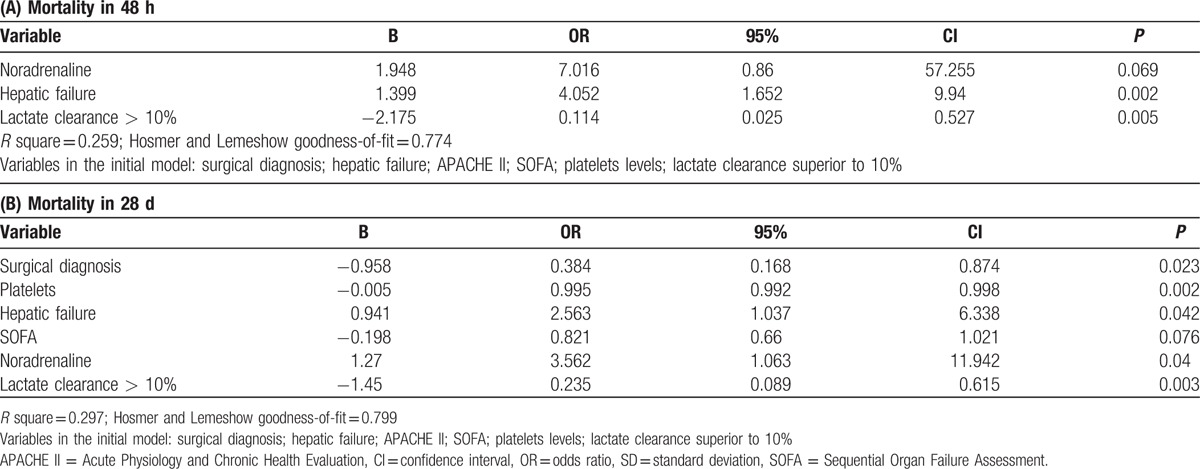
Multivariate analysis for early (A) and late (B) mortality. In these models, lactate was evaluated as lactate clearance superior to 10%.

### 3.3 ROC curve analysis

**Figure 1:**
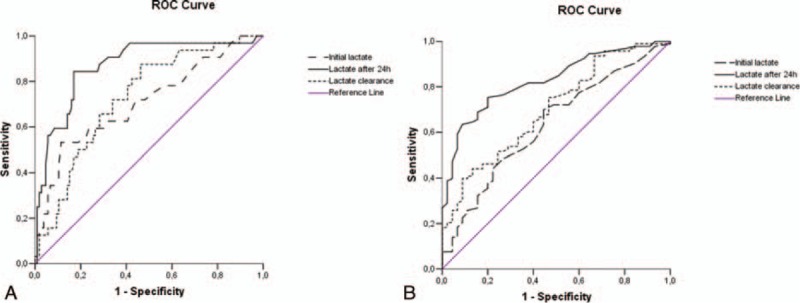
Area under the ROC curve for mortality for initial lactate, lactate 24 h after initiation of CRRT and lactate clearance for early (A) and late (B) mortality. (A) Early (48 h) mortality. AUC for initial lactate = 0.708 (95%CI = 0.599–0.817); lactate after 24 h = 0.870 (95%CI = 0.796–0.943) and for lactate clearance = 0.729 (95%CI = 0.635–0.822). (B) Late (28 days) mortality. AUC for initial lactate = 0.635 (95%CI = 0.538–0.732); lactate after 24 h = 0.828 (95%CI = 0.759–0.896) and for lactate clearance = 0.701 (95%CI = 0.611–0.791). AUC = area under the ROC curve, CI = confidence interval, CRRT = continuous renal replacement therapy, ROC = receiver operator characteristic.

Analysis of the area under the ROC curve (AUC) for early (Fig. 1A) and late (Fig. 1B) mortality demonstrated that lactate after 24 hours was superior to initial lactate, but not to lactate clearance. AUC (95%CI) for initial lactate was 0.708 (0.599–0.817) for early mortality and 0.635 (0.538–0.732) for late mortality. AUC (95%CI) for lactate after 24 hours was 0.870 (0.796–0.943) and 0.828 (0.759–0.896) for early and late mortality, respectively. AUC (95%CI) for lactate clearance was 0.729 (0.635–0.822) and 0.701 (0.611–0.791) for early and late mortality, respectively.

## 4 Discussion

In the present study, we have prospectively assessed the clinical utility of lactate levels and lactate clearance as predictive factors of early (48 hours) and late (28 days) mortality in critically ill patients with septic AKI in need of CRRT. Our results suggest that lactate levels measured 24 hours after the initiation of CRRT were strongly associated with both early and late mortality. Lactate clearance, as defined by the percentage of lactate cleared over the 24-hours period after initiation of CVVHDF, was independently associated with lower mortality.

Patients with septic AKI are generally sicker, with a higher burden of illness, and have greater abnormalities in acute physiology compared with patients with nonseptic AKI. Moreover, septic AKI is independently associated with higher odds of death and longer duration of hospitalization.^[2,3]^ However, little is known about risk-stratification biomarkers in septic AKI patients on CRRT patients and our data suggests that lactate could be a plausible candidate.

In our study general scoring systems (APACHE II, SOFA) did not discriminate prognosis; however, temporal evolution of lactate during CRRT treatment was significantly different between survivors and nonsurvivors. This finding is congruent with some data that have shown that failure to correct metabolic acidosis rapidly during CRRT is a strong predictor of mortality. For example, Page et al^[8]^ have demonstrated that metabolic acidosis was associated with increased lactate levels mainly resulting from circulatory failure. In another study, lactate was shown to be associated with early (48 hours) mortality in patients in need of CRRT by Kawarazaki et al.^[16]^ The authors suggested that lactate, among other variables, could help differentiate acute kidney injury patients who do not benefit from CRRT. Our results demonstrate that lactate is associated with mortality also in the specific subgroup of septic AKI patients and is associated with late (28 days), in addition to early (48 hours), mortality.

Lactate clearance over time has been shown to be superior to oxygen-derived variables (oxygen deliver [DO2} and oxygen consumption [VO2]) in septic shock patients.^[17]^ Our results are consistent with this view. In our study, a lactate clearance higher than 10% was associated with survival. Moreover, in multivariate analysis, lactate at 24 hours after initiation of CRRT, but not initial lactate was associated with mortality. Furthermore, analysis of the area under the ROC curve have shown that lactate at 24 hours demonstrated better prognostic value than initial lactate.

An increase in blood lactate concentration may be due to defective lactate clearance, despite normal lactate production or to lactate overproduction with more or less normal lactate clearance.^[18]^ Severe acidemia is an important factor in lactate metabolism impairment; thus, the beneficial effect of continuous venovenous hemofiltration with dialysis on hyperlactatemia probably reflects an improvement in acid–base and metabolic status (leading to enhanced lactate metabolism), rather than the direct removal of lactate by ultrafiltration and dialysis.^[18]^ Furthermore, Levraut et al^[19]^ have demonstrated that the amount of lactate removed by CVVHDF using bicarbonate-buffered fluids is negligible, as a result blood lactate concentration remains a reliable metabolic marker of tissue oxygenation.

Although considerable attention has focused on the perceived benefits of CRRT, there has been less emphasis on the possibility that it might induce and/or perpetuate global tissue hypoxia and hyperlactatemia.^[20,21]^ The intravascular volume depletion associated with excessive ultrafiltration rate could be caused by both the high rate of fluid removal required and the trans-cellular and interstitial fluid shifts caused by the rapid dialytic loss; this may jeopardize hemodynamic instability and induce tissue hypoxia and hypoperfusion.^[20,22]^ Bioincompatibility of renal replacement therapy materials potentially enhances coagulation and inflammation pathways that are already triggered. These processes result in tissue dysoxia, either from impaired microcirculatory oxygen delivery and/or from mitochondrial dysfunction.^[23,24]^ The heat loss that occurs during continuous renal replacement therapy favors the development of hypothermia. Long-term cooling has huge metabolic consequences, which increases oxygen demands, leads to vasoconstriction, inhibition of leukocyte function and coagulation.^[25,26]^

Liver failure commonly leads to major changes in acid–base balance status. Combined liver failure and acute kidney are associated with unique acid–base changes due mostly to lactic metabolic acidosis and, in smaller part, to the accumulation of unmeasured anions. This acidosis is attenuated by hypoalbuminemia, hypochloremia, and hypercapnia.^[27]^ Naka et al^[28]^ have shown that the use of CRRT does not fully correct the independent acidifying effect of liver failure on acid–base status. Increased lactate and strong ion gap values maintain a persistent base deficit despite the alkalinizing effects of hypoalbuminemia and hypochloremia. It has been suggested that lactate clearance which normally depends on liver gluconeogenesis, could depend more on oxidation in injured, post ischemic or resting tissues during stressful states including sepsis.^[29]^ Our results suggest that clearance of lactate remained strongly associated with outcome even in patients with liver failure.

Our study has several limitations. Because of the observational nature of study cohort, our findings are at best association, causation need further confirmation. Nonetheless our observation of significant correlation of lactate clearance and decrease of mortality is consistent with previous studies in different populations. We do not have data to assess how closely the care team complied with early goal direct therapy in septic shock in this group of patients before or during CVVHDF. Our database did not include data on the exact indications, rate of ultrafiltration and timing for CVVHDF. Although the same medical team cared for all patients in this study, there was an inevitable day-to-day variability in the practitioners who were in charge of the patients. These changes might have induced some variability in the quality of care, which could have caused a degree of bias in the results.

## 5 Conclusion

Lactate clearance and lactate at 24 hours after initiation of CRRT were associated with 48 hours and 28-days mortality in septic AKI patients undergoing CVVHDF. These findings suggest that lactate clearance and serial lactate measures may be more useful prognostic markers than initial lactate alone. Whether interventions during CRRT aimed to improve lactate clearance will improve outcomes remains unknown and warrants further investigation.
